# Gut microbiota and plasma metabolites in pregnant mothers and infant atopic dermatitis: A multi-omics study^☆^

**DOI:** 10.1016/j.waojou.2024.101017

**Published:** 2025-01-02

**Authors:** Bingqian Du, Aga Shama, Yi Zhang, Baolan Chen, Yongqi Bu, Pei-an Chen, Chuzhi Lin, Jie Liu, Juan Zheng, Zhenjun Li, Qingsong Chen, Yu Sun, Xi Fu

**Affiliations:** a Guangdong Provincial Engineering Research Center of Public Health Detection and Assessment, NMPA Key Laboratory for Technology Research and Evaluation of Pharmacovigilance, School of Public Health, Guangdong Pharmaceutical University, Guangzhou, 510006, Guangdong, PR China; b National Key Laboratory of Intelligent Tracking and Forecasting for Infectious Diseases, National Institute for Communicable Disease Control and Prevention, Chinese Center for Disease Control and Prevention, Beijing, 102200, PR China; c Maternity and Child Health Hospital of Baiyun District, Guangzhou, 510400, Guangdong, PR China; d State Key Laboratory of Swine and Poultry Breeding Industry, Guangdong Provincial Key Laboratory for the Development Biology and Environmental Adaptation of Agricultural Organisms, College of Life Sciences, South China Agricultural University, Guangzhou, 510642, Guangdong, PR China

**Keywords:** Fatty acids, Metabolome, Virulent factors, Flavonoids, Atopic dermatitis

## Abstract

**Background:**

Many studies reported the influence of infants' gut microbiota on atopic dermatitis (AD) postnatally, yet the role of maternal gut microbiota and plasma metabolites in infants’ AD remains largely unexplored.

**Methods:**

Sixty-three pregnant mother-infants were enrolled and followed after childbirth in Guangzhou, China. Demographic information, maternal stool and plasma samples, and records for infants’ AD were collected. Maternal gut microbiota/metabolome and plasma metabolome were profiled using shotgun metagenomics and non-targeted metabolomics. Logistic regression and multi-omics analysis were used to explore characteristic maternal gut microbiota in the AD and health groups.

**Results:**

The α-diversity of maternal gut microbiota in health group was significantly higher than AD group (Shannon diversity *P* = 0.02, Simpson diversity *P* = 0.04). Short-chain fatty acids (SCFAs) producing microorganisms, including *Faecalibacterium*, *Roseburia*, *Butyricicoccus*, and *Ruminococcus*, as well as the abundance of phenylalanine, tyrosine, and tryptophan biosynthesis pathway, were enriched in health group (LDA>2 and *P* < 0.05). Virulent factors (VFs) involved in immune modulation were enriched in the health group, while VFs involving in adhesin were enriched in the AD group (*P* < 0.05). Metabolomic analysis showed that a polyunsaturated fatty acid/linoleic acid, 13S-hydroxyoctadecadienoic, were negatively associated with AD in both the gut and plasma samples (FDR<0.05). Several other linoleic acids and flavonoids were negatively associated with AD (FDR<0.05). Neural network analysis revealed that microorganisms enriched in health group may produce these protective fatty acids.

**Conclusions:**

Our findings show that maternal gut microorganisms/metabolites and plasma metabolites during pregnancy impact subsequent pathogenesis of infants AD. This illuminates new strategies against early AD in offspring.

**Key Message**

Short-chain fatty acids/butyrate producing microorganisms in maternal gut protect against of infants’ atopic dermatitis. Higher levels of fatty acids and flavonoids/isoflavonoids in maternal plasma metabolites during pregnancy were associated with a lower atopic dermatitis risk in early life. An appropriate diet during pregnancy could be a simple strategy to mitigate atopic dermatitis in offspring. The abundance of virulence factors genes for immune modulation were higher in the health group, but virulence factors genes for adhesin were higher in the atopic dermatitis group.

## Introduction

Atopic dermatitis (AD), also known as eczema, is a skin inflammatory condition triggered by various internal and external factors. It manifests through symptoms such as erythema, papules, and itching.^1^ AD could start in infants aged 2–3 months. The prevalence of AD is high, estimated to be 10%–15% in children and up to 10% in adults.^2^ Over recent decades, the incidence rate of AD has increased significantly in developed and developing countries.^3^ Research has highlighted an "Allergic March", a natural progression that AD first manifests in infants and children followed by other allergic diseases. Approximately 40% of these children may develop food allergies, asthma, allergic rhinitis, among other conditions, which can persist into adulthood.^4^ The high prevalence of AD in infancy imposes substantial economic and psychological burden on many families. The pathogenesis of AD involves a complex interplay of various environmental, immunological, genetic, microbial and psychological factors, but the mechanism remains not fully elucidated.^5^^,^^6^

The gut microbiota of infants plays an important role in the development of the immune system and the pathogenesis of AD.^7^ Previous studies identified significant differences between the gut microbiota of AD and healthy children, mainly focusing on *Bifidobacteria* and *Clostridium*.^8^^,^^9^ A large-scale study (n = 957) showed that higher concentrations of gut *Clostridium* cluster were associated with an increased AD risk.^10^ Similarly, another study reported that the abundance of *Bacillariophyceae*, *Clostridium* and *Enterobacteriaceae* was higher in children with AD, while *Bifidobacterium* and *Lactobacillus* was lower with AD.^11^ Low microbial diversity in infants’ gut within the first month is associated with subsequent AD.^12^ Wang et al observed that decreased gut microbiota diversity at 1 week of age was closely related to AD onset within 18 months of age.^13^

Compared to extensive studies have investigated the relationship between infants' gut microbiota and AD postnatally, few longitudinal cohort studies have examined maternal gut microbiota and its relation to subsequent AD development in infants. Microbial transfer from mother to infant begins *in utero*, continues during exposure through the birth canal at birth, and further develops through breastfeeding.^14^^,^^15^ Maternal microbiota can modulate the maternal immune system through metabolite production, which may affect infant immune development by inducing interleukin-10 (IL-10) regulatory T cells.^16^ Two studies have reported the importance of maternal gut microbiota during pregnancy in shaping the infants’ immune system function and potentially influencing AD development in offspring.^17^^,^^18^ However, these studies, which used the 16S amplicon sequencing or the quantitative culture analysis on stool samples, unable to reveal key features at the microbial species and functional gene/pathway level. Additionally, there are no studies applied high-throughput metabolome techniques in maternal gut/plasma samples to explore the association between gut metabolites, plasma metabolites, and AD.

This study aims to determine whether maternal gut microbiota, metabolites, functional pathways, virulence factors (VFs) and plasma metabolites are associated with symptoms of AD in corresponding infants, based on data from sixty-three pregnant women and their infant.

## Materials and methods

### Study design

Sixty-three pregnant women were recruited between December 2021 to March 2022, and their infants were followed at least once every 3 months during the first years of life. Maternal stool and plasma samples were collected during this period. All participants signed informed consent, and personal, lifestyle and dietary information were obtained through a self-administered questionnaire. The diagnosis of AD in infants was conducted by pediatricians with specialized training in pediatric dermatology and atopic conditions.

### Collection of stool and plasma samples

Maternal plasma samples were collected at hospital laboratory during second (90.5%) or third-trimester (9.5%) pregnancy. Plasma was separated and stored at −80 °C until serological tests. Participants self-collected stool samples using collection tube, either at the hospital on the day of plasma sampling or at home within a week following plasma sampling. Participants who collected stool samples at home were required to encase the tube in a sealed bag, store it in the freezer compartment of their refrigerator, and notify our staff for collection within 24 h. All samples were transferred to laboratory within 24 h and stored at −80 °C until DNA extraction.

### Stool metagenomic sequencing and analysis

Microbial DNA was extracted from 50 mg stool samples by QIAamp DNA Stool Mini Kit (Qiagen). Following DNA extraction, the genome was fragmented, and PCR amplification was performed. The sequencing library underwent quality-checks with Qubit®3.0, sequenced using a paired-end 150 bp whole genome shotgun sequencing strategy with an insert size of 400 bp on the Illumina NovaSeq platform. Reads were assigned to samples based on barcode information. Raw data were processed with Cutadapt (v1.17) and Fastp (v0.20.0), removing low-quality sequences. Raw reads underwent processing with a 5-bp sliding window to remove low-quality sequences (Q < 20, read accuracy <99%). Only reads that were longer than 50 bp and had no ambiguous bases were kept for subsequent analyses. Human reads were removed using KneadData and BMTagger. The clean reads were *de novo* assembled using MEGAHIT (v1.0.5), and genes were predicted by MetaGenoMark (v3.25) and clustered by CD-HIT (v4.8.1) with 90% sequence identity. Feature table was imported into QIIME2 for alpha and beta diversity analysis. Significantly enriched microbial taxa were evaluated through LEfSe analysis (LDA>2). Microbial VFs were annotated using DIAMOND (v2.0.4) against the Virulence Factors of Pathogenic Bacteria database (VFDB) (v2019). We performed PERMANOVA analysis with 10,000 permutations to evaluate the differences in microbial taxonomic and functional composition (β-diversity), utilizing the Adonis function in R (v3.6.1). The co-occurrence probability of microbe-metabolite interaction was estimated using a neural network algorithm (mmvec).^19^

### Metabolomic profiling of stool and plasma samples

Chemical compounds in stool and plasma samples were assessed by untargeted LC-MS (liquid chromatography-mass spectrometry) at BioNovoGene (Suzhou, China). For each sample, 50 mg stool sample or 50 μl plasma sample was added to 0.6 mL 2-chlorophenylalanine in methanol and centrifuged at 12,000 rpm at 4 °C for 10 min. The supernatant (300 μL) was filtered through a 0.22 μm membrane. Chromatography was carried out with an ACQUITY UPLC ® HSS T3 (150 × 2.1 mm, 1.8 μm) (Waters, Milford, MA, USA), with the column maintained at 40 °C. The flow rate was set at 0.25 mL/min and the injection volume was 2 μL. The LC-ESI (+)-MS analysis used mobile phases consisting of 0.1% formic acid in acetonitrile (C) and 0.1% formic acid in water (D) with the following gradient: 0–1 min, 2% C; 1–9 min, 2–50% C; 9–12 min, 50–98% C; 12–13.5 min, 98% C; 13.5–14 min, 98-2% C; 14–20 min, 2% C. For LC-ESI (−)-MS analysis, acetonitrile (A) and 5 mM ammonium formate in water (B) were used with a similar gradient. Metabolites were detected on Orbitrap Exploris 120 (Thermo Fisher Scientific, USA). Ionization were performed with spray voltages of 3.5 kV and −2.5 kV in positive and negative modes. The sheath and auxiliary gas pressures were set to 30 and 10 arbitrary units, respectively, and the capillary temperature was set at 325 °C. The mass spectrometer operated in data-dependent acquisition mode with a scanning range of *m*/*z* 81–1000 and a mass resolution of 60,000 for MS1 and 15,000 for MS2. The normalized collision energy was set at 30%, and dynamic exclusion was applied to reduce redundant MS/MS data.

Quality control samples were prepared by pooling equal volumes of each sample and were injected periodically throughout the run to monitor the instrument's performance and stability. Blank samples were also included to detect any potential contamination. The QC samples' clustering in principal component analysis (PCA) models confirmed the method's repeatability and robustness.

Metabolite annotation was performed through a multi-step process to ensure accuracy and reliability. Initially, mass spectrometry data were processed using Compound Discoverer 3.0 (Thermo Fisher Scientific). Metabolites were tentatively identified by matching the exact mass, retention time, and fragmentation pattern against the Human Metabolome Database (HMDB) (www.hmdb.ca).^20^ Metabolite quantification and data normalization were conducted to account for batch effects and instrument variability. Normalization was performed using a dry weight correction method, where the water content of each sample was determined, and metabolite concentrations were adjusted accordingly.

### Statistical analyses

Demographic characteristics were compared using the chi-square test. Linear logistic regression analyses were performed to identify metabolites associated with AD. To minimize the impact of multiple testing in metabolite identification, we conducted a two-step analysis. First, metabolites with fold changes greater than 1.5 and P-values less than 0.05 in the Wilcoxon analysis were identified as potential candidates. These selected candidates were subjected to further analysis using a logistic regression model. The regression model was adjusted for age, BMI, family income, and parity. False Discovery Rate (FDR) correction was applied to the P-values from the regression analysis using the Benjamini-Hochberg procedure to control for multiple comparisons. Associations with FDR-corrected *P*-values less than 0.05 were considered significant. Kendall's Tau correlation analyses were performed to identify potential relations between dietary factors and each of the metabolomic factors linked to AD, and *P*-values less than 0.01 were considered significant. Statistical analyses were performed with IBM SPSS Statistics (v25.0), STATA (v16.0), and R (v4.3.1).

## Results

### General demographic characteristics

The average age of the 63 pregnant women was 29.2 years. Forty-three subjects (68.3%) had a body mass index (BMI) within the normal range (18.5 ≤ BMI<24). Twenty-two subjects (34.9%) had a history of one-time pregnancy, and 8 (12.7%) had experienced at least 2 pregnancies. Twenty-one infants (33.3%) suffer from varying degrees of AD (Table 1). Additionally, 30 subjects (47.6%) had normal labor, 8 (12.7%) underwent cesarean section, 25 (39.7%) delivered at a different hospital, 4 went into labor early, and there were 3 cases of low birth weight infants and 1 case of fetal macrosomia. However, there were no significant differences (*P* > 0.05) in mode of delivery, gender, birth weight, premature birth and mode of feeding between the AD group and the healthy group (Supplementary Table 1). Furthermore, we explored the relationship between maternal dietary and nutritional supplements during pregnancy and infant AD. Notably, only fish oil supplementation exhibited a significant difference, 8 (38.1%) mothers in the AD group supplemented with fish oil during pregnancy, in contrast to just 5 (11.9%) mothers in the health group (*P* < 0.001) (Refer to Supplementary Table 2 for additional details).

**Table 1:** Baseline characteristics of infant's mothers. Demographic characteristics were compared chi-square test.

Characteristic	Total (n = 63)	Atopic dermatitis(n = 21)	Health (n = 42)	*χ*^*2*^	*P*
Age (years)					
<35	58 (92.1)	19 (90.5)	39 (92.9)	0.109	1.000
≥35	5 (7.9)	2 (9.5)	3 (7.1)		
BMI^a^ (kg/m2)				1.870	0.429
<18.5	15 (23.8)	7 (33.3)	8 (19.0)		
18.5–24	43 (68.3)	12 (57.1)	31 (73.8)		
≥24	5 (7.9)	2 (9.5)	3 (7.1)		
Education				3.843	0.297
Below junior high school	17 (27.0)	5 (23.8)	12 (28.6)		
High school	12 (19.0)	5 (23.8)	7 (16.7)		
Junior college	20 (31.7)	4 (19.0)	16 (38.1)		
Bachelor or above degrees	14 (22.2)	6 (33.3)	7 (16.7)		
Family income (￥)				2.625	0.483
<4000	8 (12.7)	2 (9.5)	6 (14.3)		
4000 - 6000	12 (19.0)	3 (14.3)	9 (21.4)		
6000 - 10000	27 (42.9)	12 (57.1)	15 (35.7)		
≥10000	16v25.4)	4 (19.0)	12 (28.6)		
Occupation				3.544	0.198
Civil servants and employees of firms and enterprises	22 (34.9)	6 (28.6)	16 (38.1)		
Workers, farmers, and merchants	15 (23.8)	8 (38.1)	7 (16.7)		
Housewives and others	26 (41.3)	7 (33.3)	19 (45.2)		
Parity				0.341	0.873
0	33 (52.4)	11 (52.4)	22 (52.4)		
1	22 (34.9)	8 (38.1)	14 (33.3)		
2 and above	8 (12.7)	2 (9.5)	6 (14.3)		
Stool and blood collection time				0.000	1.000
Second trimester	57 (90.5)	19 (90.5)	38 (90.5)		
Third trimester	6 (9.5)	2 (9.5)	4 (9.5)		
Smoke				/	/
No	63 (100.0)	/	/		
Yes	0 (0.0)	/	/		
Secondhand smoke				1.086	0.396
No	42 (66.7)	12 (57.1)	30 (71.4)		
Yes	21 (33.3)	9 (42.9)	12 (28.6)		
Drink				/	/
No	63 (100.0)	/	/		
Yes	0 (0.0)	/	/		

a Because the number of people with BMI ≥28 was small, it was combined with the number of people with BMI ≥24.

### Characteristic microorganisms in health and AD groups

**Fig. 1:**
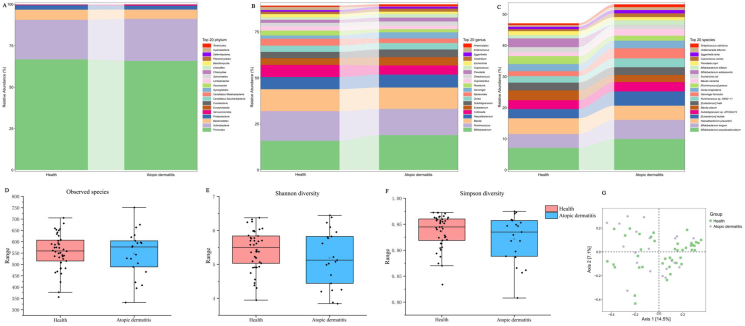
The relative abundance of taxonomical composition for major microbial phylum (A), genera (B) and species (C), observed species boxplot of alpha diversity (D), boxplot of Shannon diversity (E) and boxplot of simpson diversity (F), species PCoA diagram in the health and atopic dermatitis group (G)

The average clean data for shotgun metagenomics was 6.76 GB (Q1-Q3, 6.50–6.95 GB). In total, 2126 microbial species were characterized. The major phyla included Firmicutes (58.6% average relative abundance), Actinobacteria (16.1%), Bacteroidetes (13.5%), and Proteobacteria (8.3%) (Fig. 1A). The most abundant genera included *Bifidobacterium* (18.9%), *Ruminococcus* (15.2%), *Blautia* (11.7%), *Faecalibacterium* (6.3%), *Collinsella* (5.7%), *Eubacterium* (3.9%), and *Subdoligranulum* (3.5%) (Fig. 1B). The most abundant species were *Bifidobacterium pseudocatenulatum* (9.2%), *Bifidobacterium longum* (5.8%), *Faecalibacterium prausnitzii* (4.6%), *Eubacterium rectale* (3.9%), *Subdoligranulum* APC924/74 (2.8%), *Blautia obeum* (2.6%), *Eubacterium hallii* (2.4%), and *Ruminococcus* AM42_11 (2.4%) (Fig. 1C). The α-diversity was significantly higher in the health group than the AD group (Shannon diversity *P* = 0.024, Simpson diversity *P* = 0.039; Fig. 1D, E and F), whereas β-diversity had no significant difference between groups (*P* > 0.05, PERMANOVA; Fig. 1G).

**Fig. 2:**
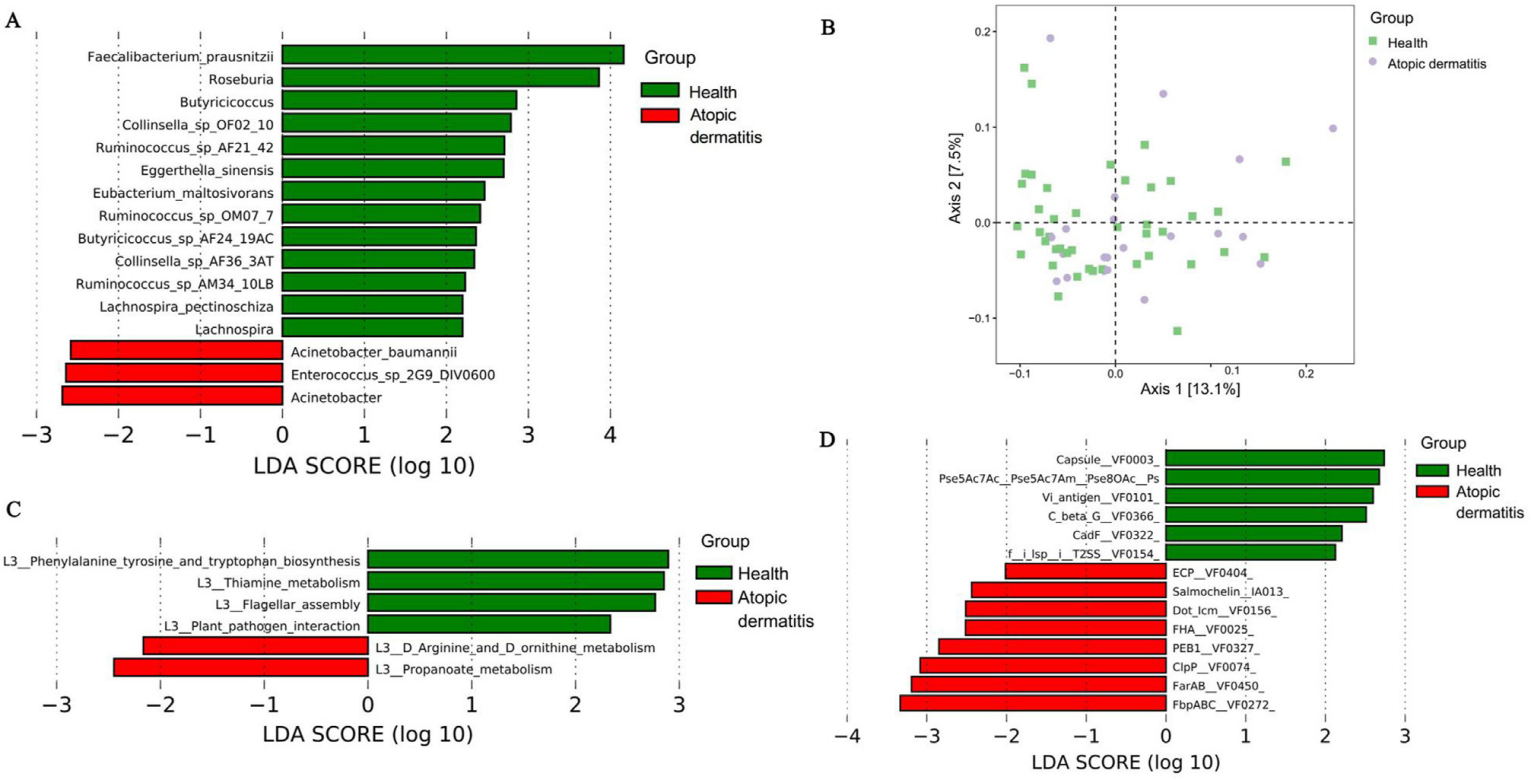
Characteristic microbial taxa (A), functional pathways (C), and virulence factor (D) in the health and atopic dermatitis group. The analysis was calculated by LEfSe (linear discriminant analysis effect size), and only taxa with LDA >2 were presented in the figure. The overall functional gene composition variation among samples is shown in the PCA analysis (B)

We further explored enriched microbial taxa in 2 groups by LEfSe analysis (Fig. 2A). *Ruminococcus* AF21_42, *Ruminococcus* OM07_7, *Ruminococcus* AM34_10LB, *Collinsella OF02_10*, *Collinsella* AF36_3AT, *F. prausnitzii*, *Eggerthella sinensis*, *Eubacterium maltosivorans*, *Butyricicoccus* AF24_19AC, *Lachnospira pectinoschiza* were enriched in the health group (LDA>2, *P* < 0.05). Interestingly, all these microorganisms are short-chain fatty acids (SCFAs)/butyrate-producing,^21^^,^^22^ suggesting a protective link against AD. *Acinetobacter baumannii* and *Enterococcus* were more abundant in the AD group (LDA>2, *P* < 0.05).

### Characteristic functional genes and VFs in health and AD groups

In total 500,743 genes were annotated against the KEGG database (Fig. 2B). Phenylalanine, tyrosine and tryptophan biosynthesis, Thiamine metabolism, Flagellar assembly, Plant pathogen interaction were enriched microbial pathway in the health group (LDA>2, *P* < 0.05; Fig. 2C). D_Arginine and D_ornithine metabolism, Propanoate metabolism were enriched in the AD group (Fig. 2C).

We performed a functional gene analysis by blasting all sequenced reads against the VFDB databases. The VFs with significantly higher abundance in health or AD group were identified (LDA score >2, *P* < 0.05; Fig. 2D). Among the health group, 3 VFs were identified as immune modulation genes, including Cyclic beta-1,2 glucan, Capsule_VF0003 and Vi_antigen_VF0101. Additionally, genes related to motility, specifically Flagella (Pse5Ac7Ac_Pse5Ac7Am_Pse8OAc_Ps), were also enriched in the health group, consistent with flagellar assembly in functional genes analysis (Fig. 2C and D). In contrast, the AD group demonstrated an enrichment of 3 VFs associated with adhesion, including *E. coli* common pilus (ECP_VF0404), Filamentous haemagglutinin (FHA_VF0025), and PEB1 (PEB1_VF0327).

### Characteristic gut and plasma metabolites in health and AD groups

**Fig. 3:**
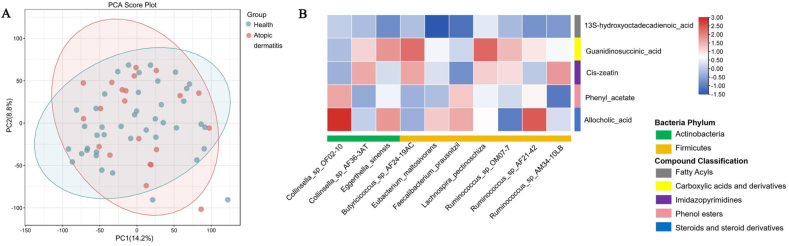
PCA diagram in Positive ion mode (A), co-occurrence probability of protective gut metabolite enriched in the health group and the featured microbial taxa in the health group (B).

Untargeted metabolomics characterized 1263 metabolites in maternal stool samples. The overall metabolite composition showed no significant difference (Fig. 3A). Potential protective gut metabolites were identified by logistic regression model (FDR<0.05, *P* < 0.05; Table 2). One fatty acid (13S-hydroxyoctadecadienoic acid), 1 carboxylic acid (guanidinosuccinic acid), 1 imidazopyrimidines (*cis*-zeatin), 1 phenol esters (phenyl acetate) and 1 steroids/bile acid (allocholic acid) were negatively/protectively associated with AD (Table 2). Three fatty acyls (dodecanoic acid, oleic acid, traumatic acid), 2 carboxylic acids [chorismate, (2S,5S)-trans-carboxymethylproline], 1 benzene (3-amino-4-hydroxybenzoate), 1 organonitrogen (Docosatetraenoyl ethanolamide), and 1 phenol (gingerol) were positively associated with AD.

**Table 2:** Logistic regression statistics for stool metabolites associated with infant atopic dermatitis. To minimize the impact of multiple testing issue, we conducted a two-step analysis. First, metabolites with fold changes >1.5 and *P* < 0.05 in the Wilcoxon analysis were identified as potential candidates. Second, these 45 selected candidates were then subjected to further analysis by logistic regression model. The regression model was adjusted for age, BMI, family income and parity. FDR correction was applied to the *P*-values in the regression analysis to lower the false discovery rate. Only associations with FDR<0.05 were shown in the table. Metabolites identified as drugs were not included in the table.

Metabolites	Compound Classification	β	95% *CI*	*P*	FDR
13S-hydroxyoctadecadienoic acid	Fatty acyls	−1.671	(-2.969, −0.375)	0.012	0.05
Guanidinosuccinic acid	Carboxylic acids and derivatives	−3.116	(-5.510, −0.721)	0.011	0.05
*Cis*-zeatin	Imidazopyrimidines	−1.435	(-2.583, −0.288)	0.014	0.05
Phenyl acetate	Phenol esters	−1.871	(-3.529, −0.213)	0.027	0.05
Allocholic acid	Steroids and steroid derivatives	−1.958	(-3.662, −0.253)	0.024	0.05
3-Amino-4-hydroxybenzoate	Benzene and substituted derivatives	2.188	(0.519, 3.856)	0.010	0.05
Chorismate	Carboxylic acids and derivatives	1.364	(0.229, 2.499)	0.018	0.05
(2S,5S)-trans-carboxymethylproline	Carboxylic acids and derivatives	1.390	(0.188, 2.591)	0.023	0.05
Dodecanoic acid	Fatty acyls	7.586	(2.170, 13.003)	0.006	0.04
Oleic acid	Fatty acyls	1.744	(0.356, 3.132)	0.014	0.05
Traumatic acid	Fatty acyls	1.484	(0.272, 2.696)	0.008	0.04
Docosatetraenoyl ethanolamide	Organonitrogen compounds	1.218	(0.243, 2.193)	0.014	0.05
Gingerol	Phenols	2.252	(0.378, 4.127)	0.019	0.05

Note: 95% Confidence interval, 95% *CI*; False discovery rate, FDR

**Fig. 4:**
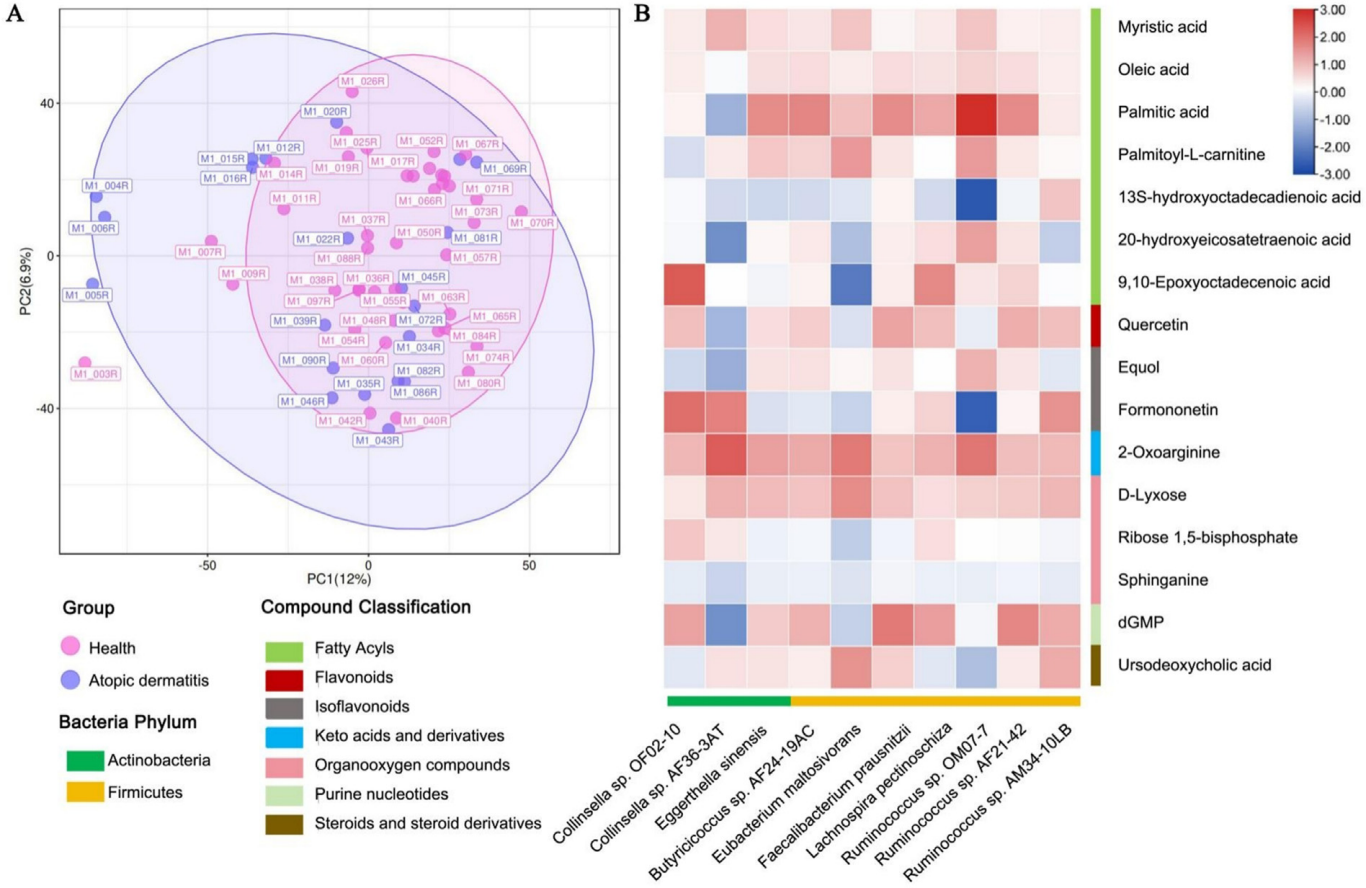
PCA diagram in Positive ion mode (A), co-occurrence probability of protective plasma metabolites enriched in the health group and the featured microbial taxa in the health group (B).

A total of 547 metabolites were identified by untargeted metabolomics in maternal plasma samples. The overall metabolite composition showed no significant difference (Fig. 4A). Seven fatty acyls, among which 3 were polyunsaturated fatty acids (PUFAs)/linoleic acids (13S-hydroxyoctadecadienoic acid, 20-hydroxyeicosatetraenoic acid, 9,10-epoxyoctadecenoic acid), showed a negative association with AD (Table 3). This finding implies that maternal linoleic acids levels might play an important role in mitigating infants’ AD. Noteworthily, 13S-hydroxyoctadecadienoic acid was the only metabolites negatively associated with AD in both the gut and plasma samples (FDR<0.05), and its abundance in gut and plasma samples was weakly correlated (*R* = 0.24, *P* = 0.054). Other potential protective metabolites included 3 organonitrogen compounds (d-lyxose, ribose 1,5-bisphosphate, and sphinganine), 3 flavonoids/isoflavonoids (quercetin, equol, and formononetin), 1 steroid (ursodeoxycholic acid), 1 keto acid (2-oxoarginine), and 1 purine nucleotides (dGMP) (Table 3). In contrast, 1 organooxygen compound (gluconic acid), 1 benzene (hordenine), 1 fatty acyls (sterculic acid), 1 indole (3-indoleacrylate), 1 phenol (3,4-dihydroxymandelic acid), 1 carboxylic acid and derivatives (l-phenylalanine), and 1 equinox (quinoline) were positively associated with AD (Table 3).

**Table 3:** Logistic regression statistics for blood metabolites associated with infant atopic dermatitis. To minimize the impact of multiple testing issue, we conducted a two-step analysis. First, metabolites with fold changes >1.5 and *P* < 0.05 in the Wilcoxon analysis were identified as potential candidates. Second, these 54 selected candidates were then subjected to further analysis by logistic regression model. The regression model was adjusted for age, BMI, family income and parity. FDR correction was applied to the *P*-values in the regression analysis to lower the false discovery rate. Only associations with FDR<0.05 were shown in the table. Metabolites identified as drugs were not included in the table.

Metabolites	Compound Classification	β	95% *CI*	*P*	FDR
Myristic acid	Fatty acyls	−4.524	(-8.076, −0.972)	0.013	0.05
Oleic acid	Fatty acyls	−1.754	(-3.255, −0.253)	0.022	0.05
Palmitic acid	Fatty acyls	−6.052	(-11.350, −0.754)	0.025	0.05
Palmitoyl-l-carnitine	Fatty acyls	−3.753	(-6.888, −0.618)	0.019	0.05
13S-hydroxyoctadecadienoic acid	Fatty acyls	−10.216	(-15.951, −4.480)	0	0
20-Hydroxyeicosatetraenoic acid	Fatty acyls	−2.766	(-5.012, −0.520)	0.016	0.05
9,10-Epoxyoctadecenoic acid	Fatty acyls	−4.991	(-8.969, −1.014)	0.014	0.05
Quercetin	Flavonoids	−2.016	(-3.579, −0.454)	0.011	0.05
Equol	Isoflavonoids	−4.014	(-6.682, −1.346)	0.003	0.04
Formononetin	Isoflavonoids	−4.308	(-7.238, −1.378)	0.004	0.04
2-Oxoarginine	Keto acids and derivatives	−13.283	(-21.702, −4.863)	0.002	0.04
d-lyxose	Organooxygen compounds	−11.793	(-21.681, −1.904)	0.019	0.05
Ribose 1,5-bisphosphate	Organooxygen compounds	−5.511	(-9.866, −1.156)	0.013	0.05
Sphinganine	Organonitrogen compounds	−8.104	(-14.450, −1.758)	0.012	0.05
dGMP	Purine nucleotides	−2.356	(-4.373, −0.339)	0.022	0.05
Ursodeoxycholic acid	Steroids and steroid derivatives	−1.939	(-3.408, −0.470)	0.010	0.05
Hordenine	Benzene and substituted derivatives	10.896	(3.136, 18.657)	0.006	0.04
l-Phenylalanine	Carboxylic acids and derivatives	10.004	(1.426, 18.582)	0.022	0.05
Quinoline	Equinoxes and derivatives	6.678	(0.977, 12.380)	0.022	0.05
Sterculic acid	Fatty acyls	4.817	(0.802, 8.833)	0.019	0.05
3-Indoleacrylate	Indoles and derivatives	4.655	(0.806, 8.504)	0.018	0.05
Gluconic acid	Organooxygen compounds	9.335	(1.984, 16.687)	0.013	0.05
3,4-Dihydroxymandelic acid	Phenols	3.513	(0.561, 6.464)	0.021	0.05

Note: 95% Confidence interval, 95% *CI*; False discovery rate, FDR

Additionally, we performed correlation analyses to investigate the potential relationships between dietary factors and each of the metabolomic factors linked to AD. Our findings suggest that fish oil supplementation during pregnancy is negatively correlated with the potential protective blood metabolites, formononetin and palmitoyl-l-carnitine, and positively correlated with the potential risk blood metabolite, gluconic acid (*P* < 0.01). Additionally, consumption of Cantonese pastries or congee during pregnancy is positively correlated with the potential risk stool metabolite dodecanoic acid (*P* < 0.01). Interestingly, dodecanoic acid is a common food additive in Cantonese pastries (Supplementary Fig. 1).

### Potential microbial source of enriched metabolites

We further explored the potential microbial source of the metabolites by neural network analysis. Several microorganisms enriched in the health group, including *Colinsella* AF36-3AT, *Butyricicoccus* AF24-19AC, *L. pectinoschiza*, and *Ruminococcus* OM07-7, showed high co-occurrence with the potential protective gut metabolites, guanidinosuccinic acid and *cis*-zeatin. Additionally, *Collinsella* OF02-10, *F. prausnitzii*, and *Ruminococcus* AF21-42, showed high co-occurrence with phenyl acetate and allocholic acid (Fig. 3B).

Moreover, several microorganisms enriched in the health group, including *E. sinensis*, *Butyricicoccus* AF24_19AC, *E. maltosivorans*, *F. prausnitzii*, *L. pectinoschiza*, and *Ruminococcus*, showed high co-occurrence with the potential protective plasma metabolites/fatty acids, including myristic acid, oleic acid, palmitic acid, palmitoyl-l-carnitine and 9,10-epoxyoctadecenoic acid. Additionally, *Collinsella* OF02-10, *E. sinensis*, *Butyricicoccus* AF24_19AC, *E. maltosivorans*, *F. prausnitzii*, *L. pectinoschiza*, *Ruminococcus* showed high co-occurrence with quercetin, equol, formononetin, 2-oxoarginine, d-lyxose, dGMP and ursodeoxycholic acid (Fig. 4B). This analysis showed that the potential gut microbiota might be mainly responsible for the potential protective metabolites production.

## Discussion

This is the first study to explore the association between maternal gut microbiota, metabolites, plasma metabolites, and infant AD using high-throughput metagenomic and metabolome techniques. Our results indicated that the enrichment or reduction of specific gut microbiota and metabolites were strongly associated with the development of AD in infants.

Specifically, we found that a high abundance of SCFAs-producing microorganisms in the maternal gut were associated with lower AD risk in infants. While the direct transfer of these correlations from mothers to infants has not been previously documented, the negative correlation between the abundance of specific microorganisms, including *Faecalibacterium*, *Roseburia*, *Ruminococcus*, *Eubacterium*, and *Lachnospira*, and the incidence of AD is well-established in both infants and adults.^21^, ^22^, ^23^, ^24^ Also, the production of SCFAs from these microorganisms are well documented. For example, *F. prausnitzii* is a major butyrate producer in human gut. *F. prausnitzii* administration in animals can reduce the levels of IL-4, IL-5, IL-13, and immunoglobulin G1, increase the proportion of Tregs, improve microbial ecological imbalance, and enhance SCFAs production.^25^ *Roseburia* not only produces SCFAs but also bacteriocin-like polypeptide with antibacterial activity, aiding in pathogen defense.^26^ An animal study showed that administering *Ruminococcus gnavus* significantly reduced AD-associated parameters and increased butyrate levels.^27^ While there are no direct links between *E. maltosivorans*, *L. pectinoschiza*, *E. sinensis*, *Collinsella* and *Butyricicoccus* and atopic diseases, their ability to produce butyrate and other SCFAs is well-documented.^28^^,^^29^ The protective effect of SCFAs include their anti-inflammatory properties, which regulate immune cell mobility and diminish the release of pro-inflammatory molecules such as TNF-α, interleukin (IL)-1β, nitric oxide, and NF-κB.^30^^,^^31^ Furthermore, butyrate can inhibit IL-2 production and lymphocyte proliferation, maintains intestinal health and immune defense, influence regulatory T cells (Tregs), and aid in immune system maturation.^32^ Additionally, AD may be associated with cutaneous barrier dysfunction. The gut microbiota is the main regulator of the Gut-Skin Axis, and a high abundance of SCFAs-producing microorganisms in the gut could be linked to integrity of cutaneous barrier.^33^, ^34^, ^35^

Besides SCFAs, PUFAs especially linoleic acids, including 13S-hydroxyoctadecadienoic, 20-hydroxyeicosatetraenoic acid, and 9,10-epoxyoctadecenoic, also showed a correlation with a lower risk of infant AD. The majority of studies indicate that fatty acids supplementation during pregnancy plays a protective role in the development of AD in early life.^36^, ^37^, ^38^ For example, alpha and gamma-linolenic acids can effectively reduce the development of ,^38^ CE and supplementation of mothers and infants with black currant seed oil, which enriched γ-linolenic acid, significantly reduces prevalence of .^39^ CE A study of 573 mother-infant pairs from a birth cohort showed that maternal erythrocyte total PUFAs were inversely associated with offspring allergies within 2 years of age.^37^ Additionally, an animal study showed that levels of linoleic acid and its metabolites were significantly decreased in AD mice, and supplementing linoleic acid significantly ameliorated AD symptoms in mice.^40^ However, 2 studies have reported that PUFAs did not have protective roles against AD symptoms.^41^^,^^42^ Our study supports the potential benefits of supplementing with SCFAs and PUFAs/linoleic acid. Future studies aimed at deciphering the complex gut equilibrium will facilitate a better preventing of AD in early life and open therapeutic opportunities.

Besides fatty acids, we also found other maternal metabolites associated with a lower risk of infants’ AD. Specifically, we discovered 2 potential protective bile acids, including allocholic acid and ursodeoxycholic acid. Oral administration of deoxycholic acid significantly improved psoriasiform dermatitis in murine model, potentially by inhibiting IL-17A production and blocking CCL20-mediated trafficking.^43^ Furthermore, potential protective metabolites included flavonoids/isoflavonoids, such as equol, formononetin, and quercetin. Flavonoids can inhibit proinflammatory chemokines, exerting beneficial effects in AD symptoms.^44^ A previous study reported that *Eggerthella*, enriched in gut of the health group, participates in flavonoid metabolism.^29^ In this study, *E. sinensis* and flavonoids/isoflavonoids have a high co-occurrence probability in mmvec analysis, consistent with the previous report. We also observed that the aromatic amino acids biosynthesis pathway (phenylalanine, tyrosine and tryptophan biosynthesis) and thiamine metabolism were enriched microbial pathway in the health group. Tryptophan is 1 of the essential aromatic amino acids involved in gut immune regulation.^9^ A previous study found that tryptophan metabolism pathway was reduced in AD patients.^45^ Additionally, thiamine can promote normal skin metabolism and prevent the occurrence of skin diseases. One study found that low intakes of thiamine was associated with skin lesion risk.^46^

In this study, VFs, such as immune modulation and flagella genes, were enriched in the health group. This enrichment suggests a possible protective role of VFs against allergic diseases, aligning with the hygiene hypothesis. The hypothesis posits that specific early-life infections, transmitted through unhygienic contact, can prevent allergies.^47^ The immunological foundation in infants primarily features a T-helper cell type (Th) 2 immune response, which is known to facilitate allergic conditions. In contrast, microbial infections can trigger the development of a Th1-type immune response, fostering a balance between Th1 and Th2 responses and, consequently, reducing the risk of allergic diseases. Therefore, flagella genes possess immunomodulatory functions and thus might be considered potential therapeutic agents for allergic diseases.^48^ In contrast, the VFs identified in the AD group were mostly related to adhesion, including *E. coli* common pilus. Adhesins increase the capacity of bacteria such as *E. coli* and *Klebsiella pneumoniae* to colonize mucin and epithelial surfaces.^49^ Previous research demonstrated that infants with AD were enriched with *E. coli* and *K. pneumoniae* in the early stages of life, along with increased adhesin gene expression,^50^ which provides a competitive advantage for the colonization of AD related bacteria. Overall, our study shows that similar to infants’ gut VFs, maternal gut VFs may also affect the occurrence of AD in infants.

We found *A. baumannii* and *Enterococcus* were enriched in the AD group. This observation aligns with previous research indicating a predominance of Enterobacteriales (34.4%) and Actinobacteria (8.8%) in the stool microbiome of AD patients.^51^ Similarly, a urine microbiome study revealed higher *Enterobacteriales* levels in AD patients compared to controls.^52^ *Enterococcus* strains can sustain and exacerbate the inflammatory response in IL-10−/− mice,^53^ while *A. baumannii* can activates the TLR2-NF-κB pathway, promoting the assembly of NLRP3 inflammasomes, intensifying the body's inflammatory response.^54^ Although progress has been made, mechanistic evidence directly linking maternal *A. baumannii* and *Enterococcus* to the development of AD in infants is yet to be explored.

There are several strengths in this study. First, this is the first multi-omics study investigating the relationship between maternal gut microbiota and metabolites, plasma metabolites and infants' AD. Gut microorganisms involved in SCFAs production, especially butyrate production and aromatic amino acids biosynthesis pathway were enriched in the health group. Furthermore, the abundance of maternal gut and blood PUFAs/linoleic acids was negatively associated with AD, and these fatty acids might be produced by protective gut microbes. These results suggest a critical role for maternal microbiota and metabolites in influencing the risk of infant AD. Second, this was a longitudinal cohort study, allowing us to explore the temporal sequence of maternal variation on infants’ health outcome. Third, shotgun metagenomics employed in this study provides a detailed and comprehensive view of gut microbiome, enabling species-level investigations and functional gene analysis, uncovering previously unrecognized connections between the maternal gut microbiome and infant AD symptoms.

There are also limitations in this study. First, the study has a relatively small sample size of 63 maternal stool and plasma samples. Multi-omics studies often involve smaller sample sizes due to the high complexity of the data being collected, the time-intensive processes involved, and the significant financial cost. To improve reliability of our results despite the smaller sample size, we employed rigorous statistical analyses, including LEfSe and logistic regression. Second, we employed untargeted metabolomics to examine maternal gut and plasma metabolites. Although this method facilitates the identification of a wide array of metabolites, it provides information only on their relative abundance, not their exact concentrations. Future research could benefit from the application of targeted metabolomics on maternal gut and plasma metabolites. Third, some plasma and stool samples were not collected on the same day, which may affect the results due to the diet variations between days. This may mainly impact metabolite results rather than gut microbiome results since previous studies showed that the gut microbiome maintains long-term stability in terms of microbial abundance within individuals.^55^

## Conclusion

In summary, our multi-omics investigation elucidates the intricate links among maternal gut microbiota, metabolites, functional pathways, virulence factors, and plasma metabolites with the development of AD in infants. We identified maternal gut microorganisms, particularly those capable of producing SCFAs and PUFAs, that may be associated with lower AD risks in offspring. A higher abundance of flavonoids/isoflavonoids in maternal plasma also may be associated with lower AD risks in early life. These insights highlight the potential of dietary interventions rich in fatty acids and flavonoids during pregnancy as a preventive strategy against infantile AD. Future research should aim to unravel the precise mechanisms through which maternal microorganisms and their metabolites mitigate AD development in offspring, paving the way for novel preventive approaches.

## Abbreviations

AD, atopic dermatitis; SCFAs, short-chain fatty acids; VFs, virulent factors; PCA, principal component analysis; HMDB, the Human Metabolome Database; FDR, False Discovery Rate; PUFAs, polyunsaturated fatty acids; VFDB, the Virulence Factors of Pathogenic Bacteria database; IL, interleukin.

## Funding

National Natural Science Foundation of China (Youth Program): 42307541; Guangzhou Science and Technology Planning Project: SL2023A003J00806.

## Availability of data and materials

The data that support the findings of this study are available from the corresponding author, Xi Fu, upon reasonable request.

## Authors’ information

Bingqian Du: dubingqian9866@163.com authors’ consent for publication; obtained, author contributions; investigation, analysis and interpretation, visualization, writing, and editing.

Aga Shama: 709108257@qq.com authors’ consent for publication; obtained, author contributions; investigation, analysis and interpretation, writing, and editing.

Yi Zhang: zhangyi62594@163.com authors’ consent for publication; obtained, author contributions; investigation, analysis, interpretation, and editing.

Baolan Chen: cbl122200@163.com authors’ consent for publication; obtained, author contributions; investigation, analysis, and interpretation.

Yongqi Bu: 2286961915@qq.com authors’ consent for publication; obtained, author contributions; investigation, analysis, and interpretation.

Pei-an Chen: cpa838229837@163.com authors’ consent for publication; obtained, author contributions; investigation, analysis, and interpretation.

Chuzhi Lin: 814680436@qq.com authors’ consent for publication; obtained, author contributions; investigation, analysis, and interpretation.

Jie Liu: landyhunter@163.com authors’ consent for publication; obtained, author contributions; investigation, interpretation, editing, and project administration.

Juan Zheng: zhengjuan320@163.com authors’ consent for publication; obtained, author contributions; investigation, interpretation, editing, and project administration.

Zhenjun Li: lizhenjun@icdc.cn authors’ consent for publication; obtained, author contributions; conceptualization, methodology, supervision, and review.

Qingsong Chen: qingsongchen@aliyun.com authors’ consent for publication; obtained, author contributions; conceptualization, methodology, supervision, review, and project administration.

Yu Sun: sunyu@scau.edu.cn authors’ consent for publication; obtained, author contributions; conceptualization, methodology, interpretation, supervision, writing, review, and editing.

Xi Fu: fuxi@gdpu.edu.cn authors’ consent for publication; obtained, author contributions; conceptualization, methodology, interpretation, supervision, writing, review, and project administration.

All authors read and approved the final version of the article.

## Ethics approval

The study protocol was approved by the Medical Ethics Review Board of the School of Public Health, Guangdong Pharmaceutical University (School of Public Health, Guangdong Pharmaceutical University, Medical Ethics [2021] No.01).

## Declaration of competing interest

The authors declare that the research was conducted in the absence of any commercial or financial relationships that could be construed as a potential conflict of interest.
