# Supplementary material for: Gut microbiota and plasma metabolites in pregnant mothers and infant atopic dermatitis: A multi-omics study
Source: World Allergy Organ J. 2025 Jan 2;18(1):101017. doi: 10.1016/j.waojou.2024.101017 (PMC11754505; doi:10.1016/j.waojou.2024.101017)
Supplement: Multimedia component 1 [file mmc1.docx]

**Supplementary Files**


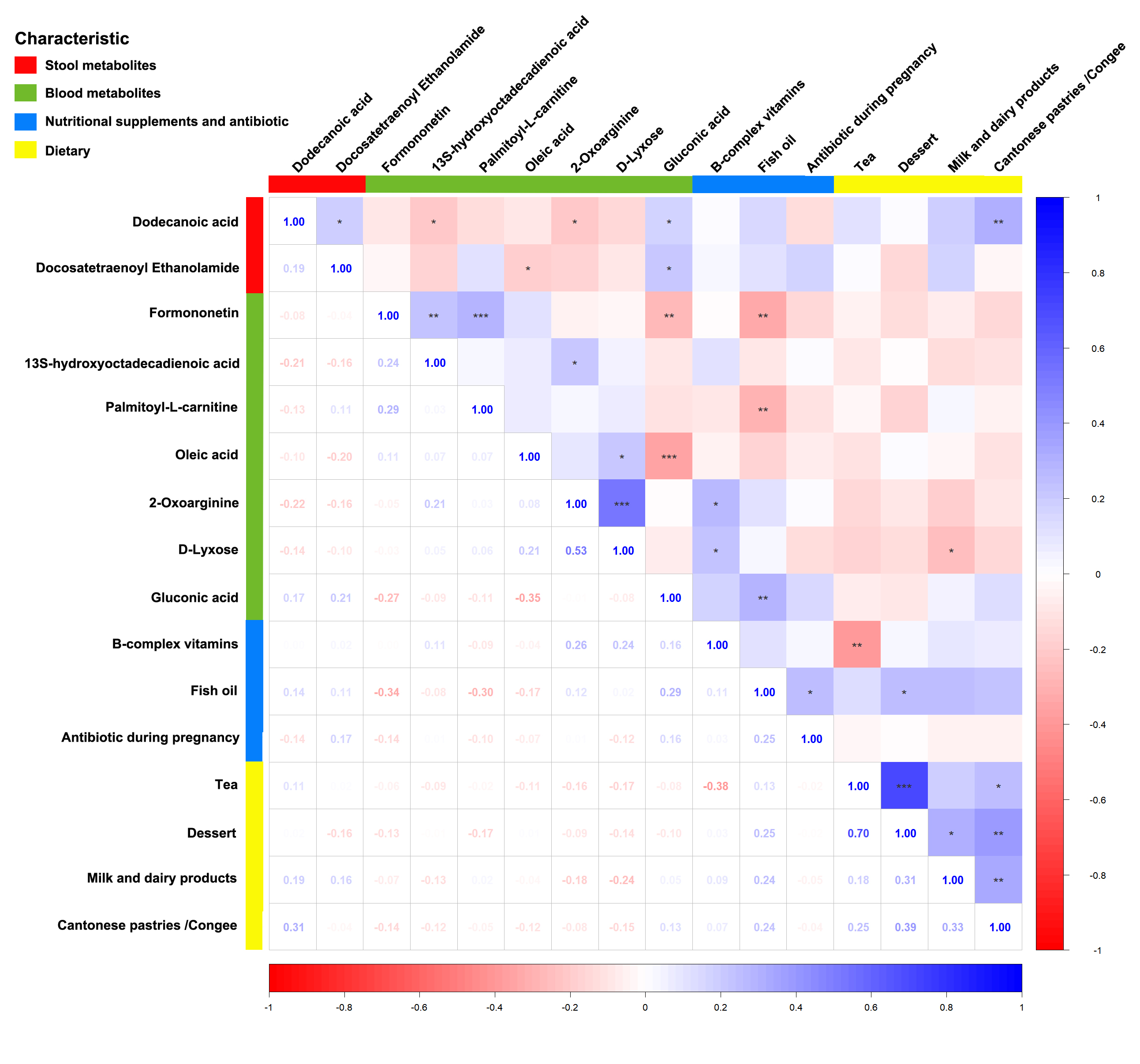


Supplementary Figure 1. Kendall's Tau correlation analysis for stool and blood metabolites associated with dietary factors. (Note: ^*^*P* < 0.05, ^**^*P* < 0.01, ^**^*P* < 0.001)

Supplementary Table 1. Delivery method, birth situation and feeding mode of infant (N=63)

| Characteristic | Total | Atopic dermatitis | Health | *χ²* | *P* |
| --- | --- | --- | --- | --- | --- |
| Delivery |  |  |  | 2.415 | 0.299 |
| Caesarean section | 8 (21.1) | 1 (8.3) | 7 (26.9) |  |  |
| Eutocia | 30 (78.9) | 11 (91.7) | 19 (73.1) |  |  |
| Gender of infant |  |  |  | 1.413 | 0.493 |
| Male | 19 (51.4) | 7 (63.6) | 12 (46.2) |  |  |
| Female | 18 (48.6) | 4 (36.4) | 14 (53.8) |  |  |
| Birth weight ^a^ |  |  |  | / | 0.287 |
| Low Birth Weight and fetal macrosomia | 4 (10.5) | 0 (0.0) | 4 (15.4) |  |  |
| Normal | 34 (89.5) | 12 (100.0) | 22 (84.6) |  |  |
| Premature birth ^a^ |  |  |  | / | 0.292 |
| No | 59 (93.7) | 21 (100.0) | 38 (90.5) |  |  |
| Yes | 4 (6.3) | 0 (0.0) | 4 (9.5) |  |  |
| Continued breastfeeding |  |  |  | 2.577 | 0.108 |
| No | 30 (47.6) | 13 (61.9) | 17 (40.5) |  |  |
| Yes | 33 (52.4) | 8 (38.1) | 25 (59.5) |  |  |
| Formula feeding |  |  |  | 1.212 | 0.271 |
| No | 24 (38.1 | 6 (28.6) | 18 (42.9) |  |  |
| Yes | 39 (61.9) | 15 (71.4) | 24 (57.1) |  |  |
| Complementary feeding |  |  |  | 2.235 | 0.135 |
| No | 22 (34.9) | 10 (47.6) | 12 (28.6) |  |  |
| Yes | 41 (65.1) | 11 (52.4) | 30 (71.4) |  |  |
| Mixture feeding |  |  |  | 1.286 | 0.257 |
| No | 21 (33.3) | 9 (42.9) | 12 (28.6) |  |  |
| Yes | 42 (66.7) | 12 (57.1) | 30 (71.4) |  |  |

^a^: Using Fisher's Exact Probability Method

Supplementary Table 2. Dietary preferences of infant’s mothers. Demographic characteristics were compared chi-square test (N=63)

| Characteristic | Total | Atopic dermatitis | Health | *χ²* | *P* |
| --- | --- | --- | --- | --- | --- |
| B-complex vitamins |  |  |  | 0.000 | 1.000 |
| No | 3 (4.8) | 1 (4.8) | 2 (4.8) |  |  |
| Yes | 60 (95.2) | 20 (95.2) | 40 (95.2) |  |  |
| Iron |  |  |  | 0.072 | 1.000 |
| No | 55 (87.3) | 18 (85.7) | 37 (88.1) |  |  |
| Yes | 8 (12.7) | 3 (14.3) | 5 (11.9) |  |  |
| Probiotics |  |  |  | / | / |
| No | 63 (100.0) | / | / |  |  |
| Yes | 0 (0.00) | / | / |  |  |
| Fish oil |  |  |  | 5.864 | **0.023** |
| No | 50 (79.4) | 13 (61.9) | 37 (88.1) |  |  |
| Yes | 13 (20.6) | 8 (38.1) | 5 (11.9) |  |  |
| Calcium |  |  |  |  |  |
| No | 54 (85.7) | 18 (85.7) | 36 (85.7) | 0.000 | 1.000 |
| Yes | 9 (14.3) | 3 (14.3) | 6 (14.3) |  |  |
| Drinking tea ^a^ |  |  |  | / | 0.108 |
| No | 61 (96.8) | 19 (90.5) | 42 (100.0) |  |  |
| Yes | 2 (3.2) | 2 (9.5) | 0 (0.0) |  |  |
| Drinking herbal tea |  |  |  | 0.258 | 1.000 |
| No | 61 (96.8) | 20 (95.2) | 41 (97.6) |  |  |
| Yes | 2 (3.2) | 1 (4.8) | 1 (2.4) |  |  |
| Burn incense ^a^ |  |  |  | / | 0.548 |
| No | 61 (96.8) | 21 (100.0) | 40 (95.2) |  |  |
| Yes | 2 (3.2) | 0 (0.0) | 2 (4.8) |  |  |
| Oral contraceptive |  |  |  | 0.046 | 1.000 |
| No | 49 (77.8) | 16 (76.2) | 33 (78.6) |  |  |
| Yes | 14 (22.2) | 5 (23.8) | 9 (21.4) |  |  |
| Pets |  |  |  | 0.000 | 1.000 |
| No | 57 (90.5) | 19 (90.5) | 38 (90.5) |  |  |
| Yes | 6 (9.5) | 2 (9.5) | 4 (9.5) |  |  |
| Antibiotics within one year before pregnancy |  |  |  | 0.463 | 0.735 |
| No | 51 (81.0) | 18 (85.7) | 33 (78.6) |  |  |
| Yes | 12 (19.0) | 3 (14.3) | 9 (21.4) |  |  |
| Antibiotics during pregnancy ^a^ |  |  |  | / | 0.333 |
| No | 62 (98.4) | 20 (95.2) | 42 (100.0) |  |  |
| Yes | 1 (1.6) | 1 (4.8) | 0 (0.0) |  |  |
| Breakfast frequency ^a^ |  |  |  | / | 0.175 |
| Every day | 51 (81.0) | 20 (95.2) | 31 (73.8) |  |  |
| 4-6 times a week | 7 (11.1) | 1 (4.8) | 6 (14.3) |  |  |
| Hardly | 5 (7.9) | 0 (0.0) | 5 (11.9) |  |  |
| Midnight snack frequency |  |  |  | 6.865 | 0.080 |
| Every day | 9 (14.3) | 4 (19.0) | 5 (11.9) |  |  |
| 4 to 6 times a week | 5 (7.9) | 4 (19.0) | 1 (2.4) |  |  |
| 1 to 3 times a week | 11 (17.5) | 2 (9.5) | 9 (21.4) |  |  |
| Hardly | 38 (60.3) | 11 (52.4) | 27 (64.3) |  |  |
| Barbecue/Seafood |  |  |  | / | / |
| No | 63 (100.0) | / | / |  |  |
| Yes | 0 (0.00) | / | / |  |  |
| Dessert ^a^ |  |  |  | / | 0.333 |
| No | 62 (98.4) | 20 (95.2) | 42 (100.0) |  |  |
| Yes | 1 (1.6) | 1 (4.8) | 0 (0.0) |  |  |
| Puffed food |  |  |  | / | / |
| No | 63 (100.0) | / | / |  |  |
| Yes | 0 (0.00) | / | / |  |  |
| Sugary drinks |  |  |  | / | / |
| No | 63 (100.0) | / | / |  |  |
| Yes | 0 (0.0) | / | / |  |  |
| Fruits and vegetables |  |  |  | 1.145 | 0.423 |
| No | 55 (87.3) | 17 (81.0) | 38 (90.5) |  |  |
| Yes | 8 (12.7) | 4 (19.0) | 4 (9.5) |  |  |
| Milk and dairy products |  |  |  | 2.333 | 0.251 |
| No | 54 (85.7) | 16 (76.2) | 38 (90.5) |  |  |
| Yes | 9 (14.3) | 5 (23.8) | 4 (9.5) |  |  |
| Hot Pot |  |  |  | / | / |
| No | 63 (100.0) | / | / |  |  |
| Yes | 0 (0.00) | / | / |  |  |
| Hamburger |  |  |  | / | / |
| No | 63 (100.0) | / | / |  |  |
| Yes | 0 (0.0) | / | / |  |  |
| Cantonese pastries /Congee |  |  |  | 3.316 | 0.166 |
| No | 57 (90.5) | 17 (81.0) | 40 (95.2) |  |  |
| Yes | 6 (9.5) | 4 (19.0) | 2 (4.8) |  |  |
| Chinese fast food |  |  |  | 0.168 | 0.762 |
| No | 47 (74.6) | 15 (71.4) | 32 (76.2) |  |  |
| Yes | 16 (25.4) | 6 (28.6) | 10 (23.8) |  |  |
| Leisure sports activities |  |  |  | 0.292 | 0.788 |
| No | 36 (57.1) | 11 (52.4) | 25 (59.5) |  |  |
| Yes | 27 (42.9) | 10 (47.6) | 17 (40.5) |  |  |
| Daily water intake |  |  |  | 2.134 | 0.339 |
| ＜1500ml | 41 (65.1) | 13 (61.9) | 28 (66.7) |  |  |
| 1500- 2000ml | 15 (23.8) | 4 (19.0) | 11 (26.2) |  |  |
| ≥ 2000ml | 7 (11.1) | 4 (19.0) | 3 (7.1) |  |  |

^a^: Using Fisher's Exact Probability Method
